# *N*-(3,5-Dichloro­phen­yl)-2-(naphthalen-1-yl)acetamide

**DOI:** 10.1107/S1600536811041468

**Published:** 2011-10-12

**Authors:** Hoong-Kun Fun, Ching Kheng Quah, B. Narayana, Prakash S. Nayak, B. K. Sarojini

**Affiliations:** a X-ray Crystallography Unit, School of Physics, Universiti Sains Malaysia, 11800 USM, Penang, Malaysia; b Department of Studies in Chemistry, Mangalore University, Mangalagangotri 574 199, Mangalore, India; c Department of Chemistry, P. A. College of Engineering, Nadupadavu, Montepadavu, P.O., Mangalore 574 153, India

## Abstract

In the title compound, C_18_H_13_Cl_2_NO, the naphthalene ring system [maximum deviation = 0.038 (4) Å] and the benzene ring form dihedral angles of 69.5 (2) and 37.2 (2)°, respectively, with the essentially planar acetamide unit [maximum deviation = 0.004 (4) Å]. The naphthalene ring system forms a dihedral angle of 52.36 (18)° with the benzene ring. In the crystal, mol­ecules are linked *via* inter­molecular N—H⋯O hydrogen bonds, forming chains along [001].

## Related literature

For the structural similarity of *N*-substituted 2-aryl­acetamides to the lateral chain of natural benzyl­penicillin, see: Mijin & Marinkovic (2006 ▶); Mijin *et al.* (2008 ▶). For the coordination abilities of amides, see: Wu *et al.* (2008 ▶, 2010 ▶). For related structures, see: Fun *et al.* (2010 ▶, 2011 ▶); Li & Wu (2010 ▶); Xiao *et al.* (2010 ▶); Praveen *et al.* (2011 ▶); Wang *et al.* (2010 ▶). For standard bond-length data, see: Allen *et al.* (1987 ▶).
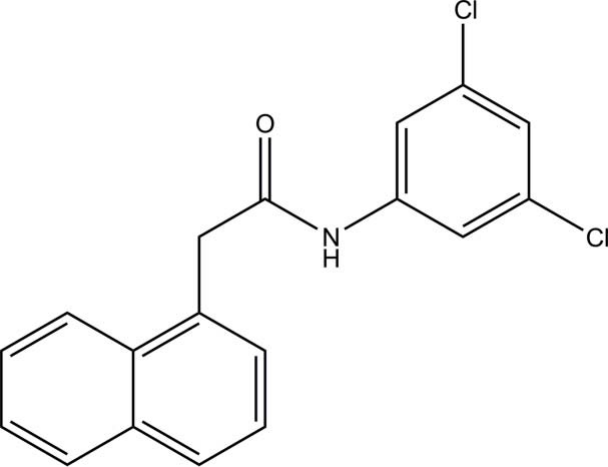


## Experimental

### 

#### Crystal data

C_18_H_13_Cl_2_NO
*M* *_r_* = 330.19
Monoclinic, 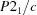

*a* = 7.8090 (14) Å
*b* = 24.811 (4) Å
*c* = 9.6783 (13) Å
β = 125.05 (1)°
*V* = 1535.1 (4) Å^3^
*Z* = 4
Mo *K*α radiation
μ = 0.42 mm^−1^
*T* = 296 K
0.38 × 0.29 × 0.06 mm

#### Data collection

Bruker SMART APEXII DUO CCD area-detector diffractometer
Absorption correction: multi-scan (*SADABS*; Bruker, 2009 ▶) *T*_min_ = 0.855, *T*_max_ = 0.974
16035 measured reflections
4453 independent reflections
2621 reflections with *I* > 2σ(*I*)
*R*_int_ = 0.051

#### Refinement

*R*[*F* ^2^ > 2σ(*F* ^2^)] = 0.074
*wR*(*F* ^2^) = 0.198
*S* = 1.03
4453 reflections
203 parameters
H atoms treated by a mixture of independent and constrained refinement
Δρ_max_ = 0.46 e Å^−3^
Δρ_min_ = −0.33 e Å^−3^

### 

Data collection: *APEX2* (Bruker, 2009 ▶); cell refinement: *SAINT* (Bruker, 2009 ▶); data reduction: *SAINT*; program(s) used to solve structure: *SHELXTL* (Sheldrick, 2008 ▶); program(s) used to refine structure: *SHELXTL*; molecular graphics: *SHELXTL*; software used to prepare material for publication: *SHELXTL* and *PLATON* (Spek, 2009 ▶).

## Supplementary Material

Crystal structure: contains datablock(s) global, I. DOI: 10.1107/S1600536811041468/lh5351sup1.cif

Structure factors: contains datablock(s) I. DOI: 10.1107/S1600536811041468/lh5351Isup2.hkl

Supplementary material file. DOI: 10.1107/S1600536811041468/lh5351Isup3.cml

Additional supplementary materials:  crystallographic information; 3D view; checkCIF report

## Figures and Tables

**Table 1:** Hydrogen-bond geometry (Å, °)

*D*—H⋯*A*	*D*—H	H⋯*A*	*D*⋯*A*	*D*—H⋯*A*
N1—H1*N*1⋯O1^i^	0.80 (4)	2.12 (4)	2.911 (4)	170 (4)

Symmetry code: (i) 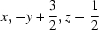.

**Fig. 1.**
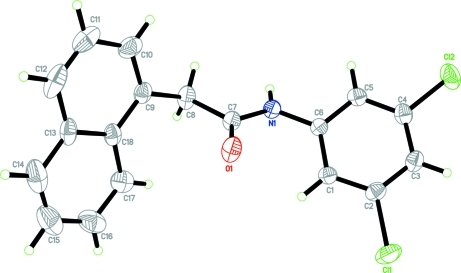
The molecular structure of the title compound showing 30% probability displacement ellipsoids for non-H atoms.

**Fig. 2.**
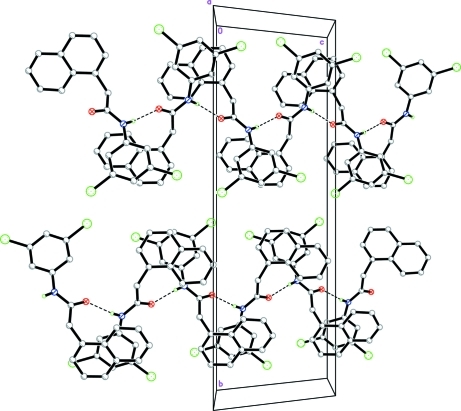
The crystal packing of the title compound, viewed along the a axis. H atoms not involved in hydrogen bonds (dashed lines) have been omitted for clarity.

## supplementary crystallographic information

### Comment

*N*-Substituted 2-arylacetamides are very interesting compounds because of their structural similarity to the lateral chain of natural benzylpenicillin (Mijin & Marinkovic, 2006; Mijin *et al.*, 2008). Amides are also used as ligands due to their excellent coordination abilities (Wu *et al.*, 2008, 2010). Crystal structures of some acetamide derivatives, viz., 2-(4-bromophenyl)-*N*-(2-methoxyphenyl)acetamide (Xiao *et al.*, 2010), *N*-benzyl-2-(2-bromophenyl)-2-(2-nitrophenoxy) acetamide (Li & Wu, 2010) and *N*-(3-chloro-4-fluorophenyl)-2- (naphthalen-1-yl)acetamide (Praveen *et al.*, 2011)have been reported. In view of the importance of amides, we report herein the crystal structure of the title compound.

The molecular structure is shown in Fig. 1. Bond lengths (Allen *et al.*, 1987) and angles are within normal ranges and are comparable to related structures (Fun *et al.*, 2010, 2011; Wang *et al.*, 2010). The naphthalene ring system (C9-C18, maximum deviation of 0.038 (4) Å at atom C9) and the benzene ring (C1-C6) form dihedral angles of 69.5 (2) and 37.2 (2)°, respectively, with the acetamide moiety (O1/N1/C7/C8, maximum deviation of 0.004 (4) Å at atom C7). The naphthalene ring system forms a dihedral angle of 52.36 (18)° with the benzene ring.

In the crystal, (Fig. 2), molecules are linked *via* intermolecular N1–H1N1···O1^i^ hydrogen bonds (Table 1) to form one-dimensional chains along [001].

### Experimental

Naphthalen-1-acetic acid (0.186g, 1 mmol) and 3,5-dichloroaniline (0.162g, 1 mmol) were dissolved in dichloromethane (20 ml). The mixture was stirred in presence of triethylamine at 273 K for about 3 h. The contents were poured into 100 ml of ice-cold aqueous hydrochloric acid with stirring, and was extracted thrice with dichloromethane. Organic layer was washed with saturated NaHCO_3_ solution and brine solution, dried and concentrated under reduced pressure to give the title compound. Single crystals were grown from toluene and acetone mixture by the slow evaporation method (*m.p.*: 422-425 K).

### Refinement

Atom H1N1 was located from the difference Fourier map and refined freely N1–H1N1 = 0.80 (4) Å]. The remaining H atoms were positioned geometrically and refined using a riding model with C–H = 0.93 or 0.97 Å and *U*_iso_(H) = 1.2 *U*_eq_(C).

### Crystal data

**Table:**

C_18_H_13_Cl_2_NO	*F*(000) = 680
*M**_r_* = 330.19	*D*_x_ = 1.429 Mg m^−^^3^
Monoclinic, *P*2_1_/*c*	Mo *K*α radiation, λ = 0.71073 Å
Hall symbol: -P 2ybc	Cell parameters from 3344 reflections
*a* = 7.8090 (14) Å	θ = 2.7–29.8°
*b* = 24.811 (4) Å	µ = 0.42 mm^−^^1^
*c* = 9.6783 (13) Å	*T* = 296 K
β = 125.05 (1)°	Plate, colourless
*V* = 1535.1 (4) Å^3^	0.38 × 0.29 × 0.06 mm
*Z* = 4	

### Data collection

**Table:**

Bruker SMART APEXII DUO CCD area-detector diffractometer	4453 independent reflections
Radiation source: fine-focus sealed tube	2621 reflections with *I* > 2σ(*I*)
graphite	*R*_int_ = 0.051
φ and ω scans	θ_max_ = 30.0°, θ_min_ = 2.7°
Absorption correction: multi-scan (*SADABS*; Bruker, 2009)	*h* = −10→8
*T*_min_ = 0.855, *T*_max_ = 0.974	*k* = −33→34
16035 measured reflections	*l* = −13→13

### Refinement

**Table:**

Refinement on *F*^2^	Primary atom site location: structure-invariant direct methods
Least-squares matrix: full	Secondary atom site location: difference Fourier map
*R*[*F*^2^ > 2σ(*F*^2^)] = 0.074	Hydrogen site location: inferred from neighbouring sites
*wR*(*F*^2^) = 0.198	H atoms treated by a mixture of independent and constrained refinement
*S* = 1.03	*w* = 1/[σ^2^(*F*_o_^2^) + (0.0681*P*)^2^ + 1.5936*P*] where *P* = (*F*_o_^2^ + 2*F*_c_^2^)/3
4453 reflections	(Δ/σ)_max_ = 0.001
203 parameters	Δρ_max_ = 0.46 e Å^−^^3^
0 restraints	Δρ_min_ = −0.33 e Å^−^^3^

### Special details

**Table:**

Geometry. All esds (except the esd in the dihedral angle between two l.s. planes) are estimated using the full covariance matrix. The cell esds are taken into account individually in the estimation of esds in distances, angles and torsion angles; correlations between esds in cell parameters are only used when they are defined by crystal symmetry. An approximate (isotropic) treatment of cell esds is used for estimating esds involving l.s. planes.
Refinement. Refinement of F^2^ against ALL reflections. The weighted R-factor wR and goodness of fit S are based on F^2^, conventional R-factors R are based on F, with F set to zero for negative F^2^. The threshold expression of F^2^ > 2sigma(F^2^) is used only for calculating R-factors(gt) etc. and is not relevant to the choice of reflections for refinement. R-factors based on F^2^ are statistically about twice as large as those based on F, and R- factors based on ALL data will be even larger.

### Fractional atomic coordinates and isotropic or equivalent isotropic displacement parameters (Å^2^)

**Table:**

	*x*	*y*	*z*	*U*_iso_*/*U*_eq_	
Cl1	0.6802 (2)	0.95869 (4)	0.44624 (14)	0.0775 (4)	
Cl2	0.27251 (17)	0.91840 (5)	−0.21419 (12)	0.0771 (3)	
O1	0.8282 (4)	0.75802 (9)	0.4568 (3)	0.0637 (7)	
N1	0.8058 (4)	0.78590 (10)	0.2249 (3)	0.0414 (6)	
C1	0.7351 (5)	0.86778 (12)	0.3249 (4)	0.0420 (6)	
H1A	0.8298	0.8575	0.4369	0.050*	
C2	0.6296 (5)	0.91611 (12)	0.2847 (4)	0.0485 (7)	
C3	0.4862 (5)	0.93244 (13)	0.1200 (4)	0.0511 (8)	
H3A	0.4155	0.9651	0.0954	0.061*	
C4	0.4521 (5)	0.89863 (13)	−0.0058 (4)	0.0488 (7)	
C5	0.5533 (5)	0.85030 (12)	0.0274 (4)	0.0440 (7)	
H5A	0.5265	0.8282	−0.0606	0.053*	
C6	0.6960 (4)	0.83471 (11)	0.1934 (3)	0.0377 (6)	
C7	0.8686 (5)	0.75141 (12)	0.3535 (4)	0.0438 (7)	
C8	0.9966 (6)	0.70397 (14)	0.3588 (5)	0.0580 (9)	
H8A	0.9423	0.6935	0.2440	0.070*	
H8B	1.1401	0.7156	0.4125	0.070*	
C9	0.9946 (5)	0.65549 (13)	0.4519 (4)	0.0484 (7)	
C10	0.8751 (6)	0.61214 (15)	0.3661 (6)	0.0665 (10)	
H10A	0.7960	0.6125	0.2488	0.080*	
C11	0.8659 (7)	0.56650 (17)	0.4478 (7)	0.0776 (13)	
H11A	0.7824	0.5373	0.3852	0.093*	
C12	0.9787 (8)	0.56556 (16)	0.6169 (8)	0.0826 (14)	
H12A	0.9710	0.5356	0.6707	0.099*	
C13	1.1104 (5)	0.60953 (14)	0.7160 (5)	0.0558 (9)	
C14	1.2266 (8)	0.6089 (2)	0.8930 (7)	0.0880 (15)	
H14A	1.2177	0.5794	0.9480	0.106*	
C15	1.3489 (8)	0.6501 (3)	0.9818 (7)	0.0930 (16)	
H15A	1.4222	0.6496	1.0987	0.112*	
C16	1.3700 (6)	0.6941 (2)	0.9035 (6)	0.0808 (13)	
H16A	1.4621	0.7217	0.9691	0.097*	
C17	1.2573 (5)	0.69744 (16)	0.7315 (5)	0.0622 (9)	
H17A	1.2705	0.7274	0.6807	0.075*	
C18	1.1179 (5)	0.65423 (13)	0.6294 (4)	0.0464 (7)	
H1N1	0.822 (5)	0.7771 (14)	0.154 (5)	0.055 (10)*	

### Atomic displacement parameters (Å^2^)

**Table:**

	*U*^11^	*U*^22^	*U*^33^	*U*^12^	*U*^13^	*U*^23^
Cl1	0.1246 (9)	0.0497 (5)	0.0709 (6)	0.0016 (5)	0.0636 (7)	−0.0107 (4)
Cl2	0.0783 (6)	0.0938 (8)	0.0501 (5)	0.0249 (5)	0.0315 (5)	0.0248 (5)
O1	0.1054 (19)	0.0600 (15)	0.0634 (15)	0.0303 (13)	0.0705 (15)	0.0228 (12)
N1	0.0576 (15)	0.0437 (14)	0.0380 (13)	0.0069 (11)	0.0363 (12)	0.0059 (10)
C1	0.0505 (16)	0.0400 (16)	0.0393 (14)	−0.0035 (12)	0.0280 (13)	0.0014 (12)
C2	0.0622 (19)	0.0408 (17)	0.0527 (18)	−0.0036 (14)	0.0388 (16)	−0.0025 (14)
C3	0.0610 (19)	0.0444 (18)	0.058 (2)	0.0096 (14)	0.0401 (17)	0.0085 (14)
C4	0.0522 (17)	0.0537 (19)	0.0464 (17)	0.0050 (14)	0.0318 (15)	0.0125 (14)
C5	0.0535 (17)	0.0475 (18)	0.0398 (15)	−0.0035 (13)	0.0319 (14)	0.0020 (12)
C6	0.0445 (15)	0.0372 (15)	0.0384 (14)	−0.0011 (11)	0.0279 (13)	0.0029 (11)
C7	0.0585 (17)	0.0451 (16)	0.0429 (15)	0.0079 (13)	0.0380 (15)	0.0071 (12)
C8	0.082 (2)	0.058 (2)	0.061 (2)	0.0252 (17)	0.057 (2)	0.0190 (16)
C9	0.0566 (18)	0.0458 (18)	0.0555 (19)	0.0134 (14)	0.0395 (16)	0.0054 (14)
C10	0.063 (2)	0.057 (2)	0.081 (3)	0.0047 (17)	0.042 (2)	−0.017 (2)
C11	0.083 (3)	0.050 (2)	0.119 (4)	−0.0045 (19)	0.069 (3)	−0.018 (2)
C12	0.106 (3)	0.047 (2)	0.141 (5)	0.020 (2)	0.098 (4)	0.020 (3)
C13	0.0616 (19)	0.054 (2)	0.069 (2)	0.0263 (16)	0.0474 (18)	0.0243 (17)
C14	0.101 (4)	0.105 (4)	0.084 (3)	0.050 (3)	0.068 (3)	0.045 (3)
C15	0.072 (3)	0.134 (5)	0.066 (3)	0.033 (3)	0.035 (2)	0.022 (3)
C16	0.047 (2)	0.114 (4)	0.064 (3)	−0.002 (2)	0.0219 (19)	−0.014 (3)
C17	0.0520 (19)	0.073 (2)	0.067 (2)	0.0016 (17)	0.0376 (18)	−0.0055 (19)
C18	0.0479 (16)	0.0506 (18)	0.0517 (17)	0.0164 (13)	0.0350 (15)	0.0117 (14)

### Geometric parameters (Å, °)

**Table:**

Cl1—C2	1.734 (3)	C9—C10	1.352 (5)
Cl2—C4	1.740 (3)	C9—C18	1.407 (4)
O1—C7	1.220 (3)	C10—C11	1.406 (6)
N1—C7	1.349 (4)	C10—H10A	0.9300
N1—C6	1.412 (4)	C11—C12	1.341 (7)
N1—H1N1	0.80 (4)	C11—H11A	0.9300
C1—C2	1.378 (4)	C12—C13	1.426 (6)
C1—C6	1.393 (4)	C12—H12A	0.9300
C1—H1A	0.9300	C13—C14	1.405 (6)
C2—C3	1.381 (5)	C13—C18	1.411 (4)
C3—C4	1.373 (5)	C14—C15	1.325 (7)
C3—H3A	0.9300	C14—H14A	0.9300
C4—C5	1.369 (4)	C15—C16	1.392 (7)
C5—C6	1.385 (4)	C15—H15A	0.9300
C5—H5A	0.9300	C16—C17	1.367 (6)
C7—C8	1.526 (4)	C16—H16A	0.9300
C8—C9	1.509 (5)	C17—C18	1.441 (5)
C8—H8A	0.9700	C17—H17A	0.9300
C8—H8B	0.9700		
			
C7—N1—C6	126.6 (2)	C10—C9—C18	118.6 (3)
C7—N1—H1N1	118 (3)	C10—C9—C8	120.5 (3)
C6—N1—H1N1	115 (3)	C18—C9—C8	120.8 (3)
C2—C1—C6	118.2 (3)	C9—C10—C11	122.4 (4)
C2—C1—H1A	120.9	C9—C10—H10A	118.8
C6—C1—H1A	120.9	C11—C10—H10A	118.8
C1—C2—C3	122.6 (3)	C12—C11—C10	119.4 (4)
C1—C2—Cl1	119.1 (2)	C12—C11—H11A	120.3
C3—C2—Cl1	118.4 (2)	C10—C11—H11A	120.3
C4—C3—C2	117.4 (3)	C11—C12—C13	121.4 (4)
C4—C3—H3A	121.3	C11—C12—H12A	119.3
C2—C3—H3A	121.3	C13—C12—H12A	119.3
C5—C4—C3	122.4 (3)	C14—C13—C18	121.0 (4)
C5—C4—Cl2	119.4 (3)	C14—C13—C12	121.5 (4)
C3—C4—Cl2	118.2 (3)	C18—C13—C12	117.5 (4)
C4—C5—C6	119.2 (3)	C15—C14—C13	120.2 (5)
C4—C5—H5A	120.4	C15—C14—H14A	119.9
C6—C5—H5A	120.4	C13—C14—H14A	119.9
C5—C6—C1	120.3 (3)	C14—C15—C16	121.3 (5)
C5—C6—N1	118.3 (3)	C14—C15—H15A	119.4
C1—C6—N1	121.4 (3)	C16—C15—H15A	119.4
O1—C7—N1	122.9 (3)	C17—C16—C15	121.0 (5)
O1—C7—C8	123.3 (3)	C17—C16—H16A	119.5
N1—C7—C8	113.8 (2)	C15—C16—H16A	119.5
C9—C8—C7	113.8 (3)	C16—C17—C18	119.7 (4)
C9—C8—H8A	108.8	C16—C17—H17A	120.2
C7—C8—H8A	108.8	C18—C17—H17A	120.2
C9—C8—H8B	108.8	C9—C18—C13	120.6 (3)
C7—C8—H8B	108.8	C9—C18—C17	122.6 (3)
H8A—C8—H8B	107.7	C13—C18—C17	116.8 (3)
			
C6—C1—C2—C3	0.7 (5)	C8—C9—C10—C11	−178.9 (3)
C6—C1—C2—Cl1	−178.5 (2)	C9—C10—C11—C12	0.2 (6)
C1—C2—C3—C4	−0.6 (5)	C10—C11—C12—C13	−0.9 (6)
Cl1—C2—C3—C4	178.6 (2)	C11—C12—C13—C14	179.2 (4)
C2—C3—C4—C5	0.4 (5)	C11—C12—C13—C18	−0.1 (5)
C2—C3—C4—Cl2	−179.9 (2)	C18—C13—C14—C15	−1.3 (6)
C3—C4—C5—C6	−0.2 (5)	C12—C13—C14—C15	179.4 (4)
Cl2—C4—C5—C6	−180.0 (2)	C13—C14—C15—C16	−1.7 (7)
C4—C5—C6—C1	0.3 (4)	C14—C15—C16—C17	3.1 (7)
C4—C5—C6—N1	−177.1 (3)	C15—C16—C17—C18	−1.4 (6)
C2—C1—C6—C5	−0.5 (4)	C10—C9—C18—C13	−2.6 (4)
C2—C1—C6—N1	176.8 (3)	C8—C9—C18—C13	177.9 (3)
C7—N1—C6—C5	−146.4 (3)	C10—C9—C18—C17	177.1 (3)
C7—N1—C6—C1	36.2 (4)	C8—C9—C18—C17	−2.4 (4)
C6—N1—C7—O1	3.1 (5)	C14—C13—C18—C9	−177.4 (3)
C6—N1—C7—C8	−176.2 (3)	C12—C13—C18—C9	1.9 (4)
O1—C7—C8—C9	23.7 (5)	C14—C13—C18—C17	2.9 (5)
N1—C7—C8—C9	−157.1 (3)	C12—C13—C18—C17	−177.9 (3)
C7—C8—C9—C10	103.2 (4)	C16—C17—C18—C9	178.8 (3)
C7—C8—C9—C18	−77.3 (4)	C16—C17—C18—C13	−1.5 (5)
C18—C9—C10—C11	1.6 (5)		

### Hydrogen-bond geometry (Å, °)

**Table:**

*D*—H···*A*	*D*—H	H···*A*	*D*···*A*	*D*—H···*A*
N1—H1N1···O1^i^	0.80 (4)	2.12 (4)	2.911 (4)	170 (4)

Symmetry codes: (i) *x*, −*y*+3/2, *z*−1/2.
